# Analysis of Model Mismatch Effects for a Model-Based Gas Source Localization Strategy Incorporating Advection Knowledge

**DOI:** 10.3390/s19030520

**Published:** 2019-01-26

**Authors:** Thomas Wiedemann, Achim J. Lilienthal, Dmitriy Shutin

**Affiliations:** 1 German Aerospace Center, 82234 Oberpfaffenhofen, Germany; dmitriy.shutin@dlr.de; 2 Centre for Applied Autonomous Sensor Systems, Örebro University, 70182 Örebro, Sweden; achim.lilienthal@oru.se

**Keywords:** robotic exploration, gas source localization, mobile robot olfaction, sparse Bayesian learning, multi-agent system, advection-diffusion model

## Abstract

In disaster scenarios, where toxic material is leaking, gas source localization is a common but also dangerous task. To reduce threats for human operators, we propose an intelligent sampling strategy that enables a multi-robot system to autonomously localize unknown gas sources based on gas concentration measurements. This paper discusses a probabilistic, model-based approach for incorporating physical process knowledge into the sampling strategy. We model the spatial and temporal dynamics of the gas dispersion with a partial differential equation that accounts for diffusion and advection effects. We consider the exact number of sources as unknown, but assume that gas sources are sparsely distributed. To incorporate the sparsity assumption we make use of sparse Bayesian learning techniques. Probabilistic modeling can account for possible model mismatch effects that otherwise can undermine the performance of deterministic methods. In the paper we evaluate the proposed gas source localization strategy in simulations using synthetic data. Compared to real-world experiments, a simulated environment provides us with ground truth data and reproducibility necessary to get a deeper insight into the proposed strategy. The investigation shows that (i) the probabilistic model can compensate imperfect modeling; (ii) the sparsity assumption significantly accelerates the source localization; and (iii) a-priori advection knowledge is of advantage for source localization, however, it is only required to have a certain level of accuracy. These findings will help in the future to parameterize the proposed algorithm in real world applications.

## 1. Introduction

### 1.1. Motivation

In modern industry, robots are mostly used to support human operators and to relieve them from physical work. However, robots are also more and more employed for additional exploration tasks. This is mostly facilitated by cheaper and easier to handle mobile robotic platforms. For example, recent developments of small Unmanned Aerial Vehicles (UAVs) enable end-users and researchers to make use of them for exploration and monitoring missions. In this context, gas exploration—the mapping of spatially distributed gas concentrations and finding potential sources—is of special interest. In many possible applications, such as in case of chemical, biological, radiological and nuclear (CBRN) events and disaster relief scenarios, increased concentrations may be of toxic and dangerous nature. When it comes to finding the sources or leaks of those toxic materials, robots have a distinct advantage with respect to safety aspects compared to human operators. Therefore, for civil protection agencies it is preferable to employ a robotic system to search for toxic gas leaks. Another field of future applications may be extraterrestrial exploration missions. As an example one may think of measuring methane concentrations on Mars [1]. In this context, sending an astronaut is not only risky but also much more expensive compared to a robotic mission.

In this paper, we consider a system of multiple robotic platforms equipped with gas sensors whose task is to autonomously find gas sources based on concentration measurements in a cooperative way. Multi-robot systems are superior to single robots: (i) redundancy makes them more robust; (ii) synergies can be exploited by sharing resources; (iii) a multi-robot system can accelerate the exploration due to distributed workload; (iv) in the context of gas exploration, a multi-robot system also shows a better sensor “aperture”; It can be considered as flexible sensor array that is capable of carrying out measurements at different locations at the same time. This is especially useful for observing dynamic phenomena like gas dispersion where estimating spatial and temporal derivatives is important.

However, a multi-robot system is harder to control compared to a single robot. A single robot might be remotely steered by one operator. Yet, steering and coordinating many robots in real time is far too complex for a single operator. Even for a team of operators it would be challenging to coordinate the robots. Thus a high level of autonomy is required for coordination and control. Furthermore, the exploration task requires more than just steering the robots to achieve a good sampling coverage of the environment. Actually, for an efficient exploration sampling locations should be selected in an intelligent way in order to collected as much information as possible with as few measurements as necessary. This requires a highly skilled operator with expert knowledge about the observed process. In this paper, we propose an exploration strategy that guides each robot to its next measurement location autonomously. This decision is based on the information previously gathered by all robots.

### 1.2. Related Work

In literature, gas source localization strategies to control mobile robots based on previously gathered measurements are called adaptive or reactive [2]. One class of adaptive strategies or algorithms to guide a robot in order to find a gas source is chemotaxis [3,4,5,6]. In this bio-inspired approach, robots follow a local gradient of the concentration. Chemotaxis assumes that a measurable quantity of gas distributions rises monotonously when approaching the source. Such a strategy has two drawbacks: First, it only works if searching for a single source. As in gradient-based optimization, the robot will get stuck at the first source, i.e., the first local maximum, and will never discover other sources. Second, turbulent airflow and gusty wind disturbs gas dispersal, which results in non-smooth chaotic instantaneous concentration distributions [7]. In those cases it is harder to estimate the gas concentration gradient and the direction and distance towards the source. As a consequence, a classic gradient-based approach may fail [1]. A possible solution is to average concentration over a certain time to smooth-out turbulence effects [8]. This necessitates sampling times starting from 30 sec upwards. These sampling times are not prohibitive in exploration scenarios. Nevertheless, considering that maybe hundreds of measurements are required to localize the sources, approaches with shorter sampling times are preferable especially when speed is a crucial factor. However, there are alternative statistical signal properties, which promise to better decode the distance to a source. For example in [9] it has been shown that the variance of the signal measured by metal oxide gas sensors is a better indicator for proximity to a source than the mean. Likewise, in [10] so called “bouts”—segments during which the gas sensor is supposed to be in contact with gas “packages”—are used to predict the distance.

In contrast to relying only on concentration measurements, incorporating other physical parameters could improve source localization strategies, for example wind or airflow (or fluid flow). This leads to the class of anemotaxis strategies (see [11,12,13]). Gas dispersion is mostly dominated by advection mechanisms. As such, anemotaxis strategies follow the airflow when triggered through detection of increased concentrations in upwind direction until they reach the source.

Furthermore, it is possible to put other assumptions or physical knowledge into the exploration strategy, for example knowledge about the gas dispersion process in form of a mathematical model. This is what we refer to as “model-based” exploration in this paper. Incorporating a mathematical process description facilitates a multitude of different approaches for exploring dynamic, distributed processes. Based on the model, information criteria, e.g., entropy [14] or the fisher information (matrix) [15], can be calculated to guide mobile robots. In the context of gas exploration, approaches that rely on a kind of information criteria are sometimes referred to as infotaxis [16].

Exploration is also related to the research field of optimal sensor placement. Here, often state space models describe the dynamics of the gas dispersion. Sensor locations can be optimized by solving observer design problems [17,18]. Furthermore, the research field of optimal experimental design [19,20] lends its methods to model-based exploration. In optimal experimental design Partial Differential Equations (PDEs) are often used as a mathematical model for the spatio-temporal dispersion process [18,21,22,23,24]. Within all these approaches the diffusion equation with advection could be repeatedly found, see [18,22,24]. The advection-diffusion PDE models the dynamic change of the gas concentrations. It considers the diffusion of gas and transportation by means of airflow. Furthermore, it considers inflow of material or gas sources as an input.

Using physical knowledge about the dispersion process to devise an exploration strategy has clear advantages. First, a model enables us to infer the location of sources or the emission rate based on concentration measurements [25]. Second as stated above, based on the model we can quantify information or uncertainty. Therefore, in this paper we develop and analyze an uncertainty/entropy based exploration strategy for a multi-robot system that makes use of a PDE that describes the gas dispersion. To this end it is helpful to formulate gas source localization in a Bayesian framework. In contrast to classical parameter identification problems in PDEs, Bayesian methods assume randomness of parameters and provide statistical information or uncertainty about them. Thus, they are particularly suitable for sensor placement and source identification in a diffusion PDE, e.g., [26,27,28]. Moreover, a Bayesian framework allows to calculate information or uncertainty values, e.g., entropy [14].

Many approaches for source identification in diffusion PDEs require an explicit or implicit assumption about the exact number of sources (e.g., [22,29]). However, we would like to avoid such a strong assumption. Instead, we assume that we do not have prior knowledge about the exact number of sources, only that they are spatially sparsely distributed. To implement this assumption we make use of Sparse Bayesian Learning (SBL) [30,31]. Further, we do not make assumptions regarding the position or strength of the sources.

### 1.3. Contributions

Incorporating model assumptions about gas dispersion into an exploration strategy seems favorable. Nevertheless, in a model-based approach for gas source localization the model and assumptions made may be wrong and this can have disastrous consequences. Since gas dispersion in reality is a very complex phenomenon, it is unclear to which degree the approximation of gas dispersion by a diffusion equation with advection is justified. A key question is: how is the source localization strategy affected by not fully fitting models and imperfect model assumptions? To overcome model mismatches, we develop a probabilistic approach that allows us to relax restrictive model assumptions in a parametrized way. Conveniently, the probabilistic formulation provides an uncertainty quantification that we then use to design an information-based exploration strategy. The analysis of the effects of relaxing the model assumptions is the main contribution of this article. This work is driven by the following research questions: If we know that our model assumptions may be wrong, what do we gain if we tell our system that the model is uncertain? How does source localization perform in case of over- or under-confidence in our model and assumptions? In this paper we study those aspects in simulations. Only there can we control the difference between simulated gas dispersion environment and the model parameters used by the exploration strategy. Furthermore, in simulations, we have the freedom to parametrize our sensor models as we want. Compared to that, realistic real world experiments do not provide the possibility to freely design model mismatch nor sensor model. Moreover, reproducibility in real world experiments is harder to achieve. For example, we have no control over wind conditions in outdoor scenarios. We would further like to remark that the simulation studies are a very important first step towards a real world application. The insight gained in the presented simulation put us in a position that allows informed adaptation and parametrization of the exploration strategy to real world conditions in the future. The simulations also provide us with an estimate of the expected performance of a model-based exploration strategy. These results are of general interest for designing or choosing a gas source localization strategy for mobile robots.

The probabilistic approach used in this work is similar to our previous work described in [32]. There we only used a diffusion PDE without an advection term. However, it is debatable whether the pure diffusion-based approximation of gas dispersion is sufficient in the presence of airflow. Thus the second contribution of this paper is an extension of the diffusion equation in [32] by an advection term. As explained in Section 2.1 the extension results in a non-linear problem. Therefore, it is necessary to apply new methods which can deal with the non-linearity in order to solve the probabilistic problem. In the scope of this paper we consider the advection, i.e., wind field, as globally a-priory known. From a practical perspective the wind may be given by weather forecast or measurements carried out before. The effect of incorporating wind information compared to pure diffusion as well as the effect of wrong wind prior information are also part of the simulation studies of this paper.

In order to design an exploration strategy for localizing the gas sources, we follow the idea of an uncertainty or entropy driven exploration strategy. This strategy guides the agents of the robotic swarm to spatial locations where currently our knowledge about the environment is low. Therefore, measurements at those locations tend to improve our overall understanding and are therefore favorable to measurements at locations, where we already have a good understanding of the process. Since the gas dispersion process is time and space variant we make use of a PDE as a mathematical model. By means of the model we are able to quantify uncertainties of parameters in different regions in our environment. The output of this approach includes an uncertainty map computed based on the concentration measurements collected so far. The *L* locations with the highest uncertainty are considered as new sampling locations (Points of Interest (POIs)). For example, *L* robots could be sent to those locations. Each robot can then take a gas concentration measurement there and a new uncertainty map is created after incorporating all new measurements. By iteratively repeating this procedure the multi-robot system can explore the gas process and is able to localize the gas sources.

The outline of this paper is as follows. Section 2.1 describes the advection-diffusion PDE used as physical model for gas dispersion, followed by Section 2.2 where we transfer the PDE into a Bayesian formulation. In Section 2.3 it is shown how the Bayesian problem can be solved, i.e., how the posterior probability of the model parameters can be calculated. Based on the posterior in Section 2.4, the uncertainty map is calculated which is the foundation for exploration. The simulation studies are described in Section 3, where we first present our simulation environment before we analyze the different model assumptions and model parameter. In Section 4 we summarize and discuss the results.

## 2. Model Based Exploration

### 2.1. Gas Dispersion PDE

In general gas dispersion can be rather complex and chaotic. However, the dynamic and spatial behavior of a gas dispersion process can be mathematically approximated by a PDE. For our purpose, we need a model with not too high complexity to be able to do all calculations online, since our exploration strategy requires an adaptive reaction to measurements. Therefore, we approximate the dynamics by a diffusion equation with advection term restricted to the planar case. Although gas dispersion takes place in 3D, a 2D approximation is expedient for certain types of applications. We consider the case of ground-based robots, which are able to sample gas concentration only in a plane close to the ground. Furthermore, we assume that sources are also close to the ground and the gas of interest is heavier than air. As a real world disaster scenario one may think of leaking barrels or pipelines. Close to the ground plane, we consider the airflow in vertical direction as small and negligible compared to the horizontal component. Moreover, the 2D approximation reduces the computational complexity which is important from a practical implementation perspective. Based on these considerations, we have chosen the 2D diffusion-advection equation as a mathematical model to approximate the gas dispersion.

In the following we use f(x,t) to describe the gas concentration at point x and time *t* in our exploration area $\Omega \subset R^{2}$. Furthermore, function u(x,t) models the spatial sources’ strength, or inflow, and $v\left(x,t\right)\in R^{2}$ is a vector function that describes the wind velocity field at a given location and time. Furthermore, we assume that this velocity field is divergence free, i.e., we assume there are no sources of airflow (e.g., fans, ventilation etc.) in the environment. Based on this notation our advection-diffusion PDE is given by:

(1) $$\frac{\partial f(x,t)}{\partial t}-\kappa \nabla^{2}f\left(x,t\right)+v{\left(x,t\right)}^{T}\nabla f\left(x,t\right)=u\left(x,t\right),\;x\in \Omega ,\;t\in R_{+}.$$

The factor κ before the Laplace operator ($\nabla^{2}$) is the diffusion coefficient. The corresponding term $\kappa \nabla^{2}f\left(x,t\right)$ models the dispersion of gas based on diffusion. In addition, the advection term $v{\left(x,t\right)}^{T}\nabla f\left(x,t\right)$, describes transportation of gas caused by airflow.

To complete the definition of the model, we also need to specify boundary and initial condition for the considered problem. To this, let ∂Ω⊂Ω define a boundary of the exploration area Ω. Then, we assume that

(2) $$\begin{array}{c} f(x,t)=0,\;\mathrm{for}\;x\in \partial \Omega ,t\in R_{+},\;\;\;\mathrm{and}\\ f(x,0)=0,x\in \Omega , \end{array}$$

which is equivalent to assuming an open field scenario with the gas concentration vanishing at the boundary and with zero initial gas concentration.

From the exploration perspective, our goal is to estimate the unknown functions f(x,t), v(x,t) and u(x,t) using measurement samples. We assume that the concentrations can be measured directly using an appropriate sensor. The scalar measurements y(x,t) can be mathematically modeled by means of a functional m(·) as

(3) y(x,t)=m(f(x,t))+ξ(x,t),

where ξ(x,t) is an additive measurement noise process. We will further assume that ξ(x,t) is white and normally distributed.

Let us stress that since both f(x,t) and v(x,t) are considered as unknown, Equation (1) is a non-linear PDE with a bilinear structure due to the scalar product $v{\left(x,t\right)}^{T}\nabla f\left(x,t\right)$.

In general the PDE in Equation (1) is difficult to handle analytically. Therefore, numerical approximation methods are needed, such as Finite Volume Method (FVM) or Finite Element Method (FEM). These imply a discretization of the space- and time-continuous representations of Equations (1)–(3). Essentially, the discretization depends on the numerical methods. For example in FVMs, continuous functions are approximated by dividing the domain Ω into small cells and assuming a constant value in each cell. When applying FEMs, the domain Ω is likewise divided into small elements, e.g., triangles, however, parametrized functions are used to approximate the original function profile in each element. Similar to the space domain, the time domain can be approximated at discrete time stamps $n\cdot T_{s};n=0,1,2,\ldots$; here, $T_{s}$ is the time between two time stamps. In general, the approach we present in this article is independent of the method used for the numerical approximation. So we treat the numerical approximation from a black box perspective, which turns the considered continuous functional Equation (1) into a system of algebraic equations.

From the assembly step of classical FEM or FVM software tools we gain the matrices D, $G_{1}$, and $G_{2}$. Based on them we can cast the PDE in Equation (1) into the following algebraic equations:

(4) $$\frac{f[n]-f[n-1]}{\Delta t}-\kappa D\cdot f\left[n\right]+v_{1}\left[n\right]\circ G_{1}\cdot f\left[n\right]+v_{2}\left[n\right]\circ G_{2}\cdot f\left[n\right]-u\left[n\right]=:r\left[n\right].$$

In our case, we will use FENICS [33] and a FEM approach for this purpose. We make use of a rectangular mesh, with uniform distributed nodes and linear test and trial functions. The symbol ∘ in Equation (4) represents the Hadamard product, and vectors f[n], u[n], $v_{1}\left[n\right]$ and $v_{2}\left[n\right]$ represent the space-discretized concentration distribution, source distribution and individual wind field components in $x_{1}$ and $x_{2}$ direction, respectively. Depending on the discretization each vector contains *C* elements. Note that, loosely speaking, matrices D, $G_{1}$, and $G_{2}$ can be interpreted as discrete counterparts of the differential operators used in the original PDE Equation (1). They are all in $R^{C\times C}$. Also, in Equation (4) we explicitly introduce a variable r[n] as the residuals of the algebraic system of equations. At present, we note that with r[n]=0 we recover the discretized version of the original model Equation (1).

In a similar fashion boundary and initial conditions in Equation (2), as well as the measurement model in Equation (3) need to be cast into a discrete setting. Following a similar argumentation as above, Equation (2) can be represented as

(5) $$R_{\mathrm{Dirichlet}}f\left[n\right]=0,\;\;\mathrm{and}\;f\left[0\right]=0,$$

where the matrix $R_{\mathrm{Dirichlet}}\in R^{C\times C}$ models the discretized version of the boundary condition f(x,t)=0,x∈∂Ω.

By means of a selection matrix M[n] in $R^{L\times C}$ that is picking *L* specific elements from f[n] to reflect the location of the *L* measurement, the numerical approximation turns our measurement model into:

(6) y[n]=M[n]f[n]+ξ[n].

### 2.2. Probabilistic Modeling of a PDE

In a classical deterministic approach, the estimation of f[n], u[n], $v_{1}\left[n\right]$ and $v_{2}\left[n\right]$, could be achieved via a numerical optimization. More precisely, we would find the solution by minimizing the residuals r[n] subject to our boundary and initial constraints. The variables are thus treated as deterministic unknowns. In contrast, in a probabilistic framework the variables of interest are considered as random variables that are described by corresponding Probability Density Functions (PDFs). As a result, additional statistical information about the variables can be computed, such as second order moments or percentiles. This information can be exploited to quantify the uncertainty of the variables; it builds the basis for our exploration strategy that we will discuss later in the text. Furthermore, the probabilistic approach enables us to plug in additional prior information like our assumption that the sources are only sparsely distributed in the spatial domain. In the following we show how our system of equations, measurements and prior assumptions can be translated into a probabilistic formulation.

In a first step we relax Equation (4) by treating r[n] as statistically independent random variables. We allow r[n] to deviate from zero with a certain precision $\tau_{s}$. This assumption is supported by a general property of a PDE that essentially describes differential, i.e. local both in time and space, behavior of quantities. As a result, from Equation (4) we can conclude that

(7) $$p\left(f\left[n\right]|v_{1}\left[n\right],v_{2}\left[n\right],u\left[n\right],f\left[n-1\right]\right)\propto e^{-\frac{\tau_{s}}{2}{∥r[n]∥}^{2}}.$$

This PDF models the probability of the concentration distribution f[n] at time stamp *n* given the components of the wind velocity field $v_{1}\left[n\right]$ and $v_{2}\left[n\right]$, the source distribution u[n] and the concentration distribution f[n−1] of the last time stamp. In a similar fashion, we assume that boundary conditions in Equation (5) likewise stochastically deviate from 0 with precision $\tau_{s}$.

Furthermore, we also cast the measurement equation in a probabilistic setting. From Equation (6) we can construct the gas concentration likelihood likelihood function as.

(8) $$p\left(y\left[n\right]|f\left[n\right]\right)\propto e^{-\frac{\tau_{m}}{2}{∥M[n]\cdot f[n]-y[n]∥}^{2}},$$

where $\tau_{m}$ models the precision of the measurement noise.

Next, we represent wind field parameters in a probabilistic setting. To this end we assume a prior distribution for wind components $p\left(v_{1}\left[n\right],v_{2}\left[n\right]\right)=p\left(v_{1}\left[n\right]\right)p\left(v_{2}\left[n\right]\right)$. The horizontal components of the wind are treated as independent, normally distributed with means $\mu_{v_{1}}$ and $\mu_{v_{2}}$ respectively and a common precision $\tau_{v}$:

(9) $$p\left(v_{1}\left[n\right],v_{2}\left[n\right]\right)\propto e^{-\frac{\tau_{v}}{2}{∥\mu_{v_{1}}-v_{1}\left[n\right]∥}^{2}-\frac{\tau_{v}}{2}{∥\mu_{v_{2}}-v_{2}\left[n\right]∥}^{2}}.$$

Parameters $\mu_{v_{1}}$ and $\mu_{v_{2}}$ specify the dominant wind velocity in the corresponding directions; the role of $\tau_{v}$ is to capture the variability of wind both in time and space. Of course, more sophisticated and more realistic distributions for the wind field are conceivable [34]. This has to be investigated further in future work.

In this work, we are modeling the prior assumption that the sources are sparsely distributed in the environment. To this end, we appeal to SBL methods. SBL is realized by imposing a hierarchical factorized prior $p\left(u\left[n\right],\gamma \left[n\right]\right)=\prod_{c=1}^{C}p\left(u_{c}\left[n\right]|\gamma_{c}\left[n\right]\right)p\left(\gamma_{c}\left[n\right]\right)$ on u[n] [30,31]. The hyper-parameter γ is also treated as a random variable and has to be estimated as well. The product $p\left(u_{c}\left[n\right]|\gamma_{c}\left[n\right]\right)p\left(\gamma_{c}\left[n\right]\right)$ builds a so called Gaussian scale mixture, where $p(u_{c}\left[n\right]|\gamma_{c}\left[n\right])$ is a zero-mean Gaussian PDF with a random precision $\gamma_{c}\left[n\right]$. Clearly, higher values of the hyper-parameter γ[n] will drive the corresponding a posterior values of u[n] toward zero. The hyper-prior $p(\gamma_{c}\left[n\right])$ is selected as a Gamma distribution $G(\gamma_{c}\left[n\right]|a,b)$, c=1,…,C with fixed hyper-prior parameters *a* and *b*. It was shown [30] that such hierarchical combination leads to an effective prior

(10) $$\begin{array}{c} p(u_{c}\left[n\right])=\int p\left(u_{c}\left[n\right]|\gamma_{c}\left[n\right]\right)p\left(\gamma_{c}\left[n\right]\right)d\gamma_{c}\left[n\right]=\\ \;\;\;=\int N\left(u_{c}\left[n\right]|0,\gamma_{c}{\left[n\right]}^{-1}\right)G\left(\gamma_{c}\left[n\right]|a,b\right)d\gamma_{c}\left[n\right], \end{array}$$

which turns out to be a Student-t distribution. The Student’s-t distribution puts more probability mass on the axes in the states space of u[n]. Finally, we note that it is common in a SBL framework to select a=0 and b=0, which leads to the sparsest solution. This choice results in a non-informative hyper-prior $p\left(\gamma_{c}\left[n\right]\right)\propto \gamma_{c}{\left[n\right]}^{-1}$, which nonetheless leads to a proper posterior distribution of source signals u[n]. It is the same choice we made in our previous work [32].

We are now ready to outline the inference approach used in our work. We define $Y=[y{\left[1\right]}^{T},\ldots ,y{\left[n\right]}^{T}]$ as a set of all observed measurement data up to a time instance *n*. To make the inference tractable, we will restrict our analysis to a single time instance *n*. Using Bayes theorem we get the posterior PDF

(11) $$\begin{array}{c} p(f\left[n\right],v_{1}\left[n\right],v_{2}\left[n\right],u\left[n\right],f\left[n-1\right]|Y\left[n\right])\propto \\ p\left(y\left[n\right]|f\left[n\right]\right)p\left(f\left[n\right]|v_{1}\left[n\right],v_{2}\left[n\right],u\left[n\right],f\left[n-1\right]\right)\\ p\left(v_{1}\left[n\right],v_{2}\left[n\right]\right)p\left(u\left[n\right]\right)p\left(f\left[n-1\right]\right|Y[n-1]). \end{array}$$

Notice that the posterior in Equation (11) is defined over two successive concentration functions f[n] and f[n−1]. The reason for this is a temporal interdependence between concentration snapshots that cannot be easily resolved. As a possible solution we include in Equation (11) the term p(f[n−1]|Y[n−1]), which summarizes the concentration value at the previous time step. The latter PDF can be computed from the posterior Equation (11) obtained at the time step n−1 and appropriately marginalized as follows:

(12) $$\begin{array}{c} p(f[n-1]|Y[n-1])=\\ \;\;\;=\int \cdots \int p(f\left[n-1\right],v_{1}\left[n-1\right],v_{2}\left[n-1\right],u\left[n-1\right],f\left[n-2\right]|Y\left[n-1\right])\\ \;\;\;\;\times dv_{1}\left[n-1\right]dv_{2}\left[n-1\right]du\left[n-1\right]df\left[n-2\right]. \end{array}$$

In this way, the estimation of the parameters of interest for a single time step can be implemented similar to a filtering procedure.

Despite the restriction of the inference problem to a single time interval, Equation (11) remains intractable. Also, marginalization in Equation (12) is in general difficult due to the non-linearity of the PDE. As a remedy, we appeal to the variational Bayesian inference methods [35,36], as explained in the following section.

### 2.3. Variational Inference

In order to make use of the probabilistic framework, we have to calculate the posterior PDF in Equation (11). This is necessary to design the exploration strategy in Section 2.4. In general this posterior is hard to calculate, because of the non-linearity introduced in Equation (4) and due to the form of the used hierarchical prior. Therefore, we make use of variational inference techniques to approximate the posterior.

To simplify the following notation, let us introduce auxiliary variables $w\left[n\right]={\left[f{\left[n\right]}^{T},v_{1}{\left[n\right]}^{T},v_{2}{\left[n\right]}^{T},f{\left[n-1\right]}^{T}\right]}^{T}$ and $\theta \left[n\right]={\left[w^{T},u{\left[n\right]}^{T}\right]}^{T}$. We will also skip an explicit dependency of variables on the time index *n* to further simplify notation. Now, consider the evidence of the model p(Y):

(13) p(Y)=∫p(Y,θ,γ)dθdγ.

The key idea of a variational Bayesian inference is to introduce a proxy distribution q(θ,γ) to approximate the posterior of interest as follows [36]:

(14) $$\begin{array}{c} \log p\left(Y\right)=\log\int q\left(\theta ,\gamma \right)\frac{p(Y,\theta ,\gamma )}{q(\theta ,\gamma )}d\theta d\gamma \\ \geq \int q\left(\theta ,\gamma \right)\log\frac{p(Y,\theta ,\gamma )}{q(\theta ,\gamma )}d\theta d\gamma =L\left(q\left(\theta ,\gamma \right)\right). \end{array}$$

The difference between the true log marginal probability and the lower bound L(q(θ,γ)) is known to be the Kullback-Leibler divergence of q(θ,γ) and the posterior (11):

(15) $$KL\left(q||p\right)=-\int q\left(\theta ,\gamma \right)\log\frac{p(\theta ,\gamma |Y)}{q(\theta ,\gamma )}d\theta d\gamma .$$

Since logp(Y) is a constant quantity for given observations, by maximizing the lower bound L(q(θ,γ)) we minimize the Kullback-Leibler divergence and q(θ,γ) will become close to the desired posterior p(θ,γ|y).

In order to solve the variational problem, i.e., maximizing L with respect to q(θ,γ) we make use of the mean field approximation:

(16) $$q\left(\theta ,\gamma \right)=q_{\theta }\left(\theta \right)\prod_{i}^{C}q_{\gamma_{i}}\left(\gamma_{i}\right).$$

In SBL the approximated PDFs $q_{\theta }$ and $q_{\gamma_{j}}$ are chosen from conjugate families. This choice ensures that during the updates the distributions keep their representation and analytical tractability of the inference expression. Classically in SBL, the posterior would be a combination of a Normal distribution and a Gamma distribution. We will make the following choices for $q_{\theta }$ and $q_{\gamma_{j}}$, j=1,…,C (see also [36]):

(17) $$\begin{array}{c} q_{\theta }\left(w,u\right)=N\left(\theta |\mu_{\theta },\Sigma_{\theta }\right)=N\left(\left[\begin{array}{c} w\\ u \end{array}\right]\left|\left[\begin{array}{c} \mu_{w}\\ \mu_{u} \end{array}\right]\right.,\left[\begin{array}{cc} \Sigma_{w,w} & \Sigma_{w,u}\\ \Sigma_{u,w} & \Sigma_{u,u} \end{array}\right]\right). \end{array}$$

(18) $$\begin{array}{c} q_{\gamma_{j}}\left(\gamma_{j}\right)=G\left(\gamma_{j}|a_{j},b_{j}\right),\;j=1\ldots C. \end{array}$$

In Equation (17) $\Sigma_{\theta }$ is in $R^{5C\times 5C}$, obviously $\Sigma_{w,w}\in R^{4C\times 4C}$ and $\Sigma_{u,u}\in R^{C\times C}$.

Now, using Equation (16), we iteratively maximize L by updating each factor of q(θ,γ) as follows [36]:

(19) $$q_{\theta }\left(w,u\right)\leftarrow \frac{1}{\alpha }exp\left({\langle \log p(y,\theta ,\gamma )\rangle }_{q_{\gamma_{i}}|i=1\ldots C})\right)$$

(20) $$q_{\gamma_{j}}\left(\gamma_{j}\right)\leftarrow \frac{1}{\beta }exp\left({\langle \log p(y,\theta ,\gamma )\rangle }_{q_{\theta },q_{\gamma_{i}}|i=1\ldots C,i\neq j}\right),\;j=1\ldots C,$$

where ${\langle a(x)\rangle }_{b(x)}$ stands for an expectation of a function a(x) with respect to the PDF b(x), and α and β are normalization constants.

Let’s start to apply the update rules to our problem. In our case we obtain

(21) $$\begin{array}{c} q_{\theta }\left(w,u\right)\leftarrow \frac{1}{\alpha }exp\left({\langle \log(p(y|w)p(w|u)p(u|\gamma )p(\gamma ))\rangle }_{G(\gamma_{i}|a_{i},b_{i})|i=1\ldots C}\right), \end{array}$$

which requires evaluating

(22) $$\begin{array}{c} {\hat{q}}_{\theta }\left(w,u\right)=exp\left({\langle \log\left(p\left(y|w\right)p\left(w|u\right)\right)-\sum_{c}\gamma_{c}u_{c}^{2}-\sum_{c}\log\frac{1}{\gamma_{c}}\rangle }_{G(\gamma_{i}|a_{i},b_{i})|i=1\ldots C}\right). \end{array}$$

To compute the expectations we make use of the following facts

$$\begin{array}{cc} {\langle \log(p(y|w)p(w|u))\rangle }_{G(\gamma_{i}|a_{i},b_{i})|i=1\ldots C} & =\log(p(y|w)p(w|u))\\ {\langle \sum_{c}\gamma_{c}u_{c}^{2}\rangle }_{G(\gamma_{i}|a_{i},b_{i})|i=1\ldots C} & =\sum_{c}\frac{a_{c}}{b_{c}}{\left(u_{c}\right)}^{2}\\ {\langle \sum_{c}\log\frac{1}{\gamma_{c}}\rangle }_{G(\gamma_{i}|a_{i},b_{i})|i=1\ldots C} & =const.\;\mathrm{with}\;\mathrm{respect}\;\mathrm{to}\;w\;\mathrm{and}\;u. \end{array}$$

This results in

(23) $$\begin{array}{c} {\hat{q}}_{\theta }\left(w,u\right)\propto p\left(y|w\right)p\left(w|u\right)\prod_{c}N\left(u_{c}|0,{\left(\frac{a_{c}}{b_{c}}\right)}^{-1}\right). \end{array}$$

Unfortunately, because of the non-linearity in Equation (4), ${\hat{q}}_{\theta }\left(w,u\right)$ is not a normal distribution. Essentially, it does not have to be a distribution at all. The factor $q_{\theta }\left(w,u\right)$ in Equation (17) is, however constrained to be normal. Therefore, we find moments of $q_{\theta }\left(w,u\right)$ so as to minimize the Kullback-Leibler divergence from $q_{\theta }\left(w,u\right)$ to ${\hat{q}}_{\theta }\left(w,u\right)$. This can be achieved by setting the mean of $q_{\theta }\left(w,u\right)$ to the maximizer of ${\hat{q}}_{\theta }\left(w,u\right)$ with respect θ, and covariance of $q_{\theta }\left(w,u\right)$ to the curvature of ${\hat{q}}_{\theta }\left(w,u\right)$ around the maximizing value. For this task we use a numerical solver. In particular, we use the Newton-CG optimization implementation in scipy [37]. Furthermore, from a numerical perspective it makes sense to consider the optimization in the log domain as follows:

(24) $$\mu_{\theta }=\arg\;\max_{\theta }\log{\hat{q}}_{\theta }\left(\theta \right).$$

To estimate the covariance matrix $\Sigma_{\theta }$ we compute the inverse of the Hessian matrix evaluated at $\mu_{\theta }$:

(25) $$\begin{array}{c} \Sigma_{\theta }={\left[-\frac{d^{2}{\hat{q}}_{\theta }\left(\theta \right)}{d\theta_{i}d\theta_{j}}{|}_{\mu_{\theta }}\right]}^{-1}. \end{array}$$

Now, we proceed similarly with factors $q_{\gamma_{j}}$, j=1,…,C. We compute

(26) $$q_{\gamma_{j}}\left(\gamma_{j}\right)\leftarrow \frac{1}{\beta }exp\left({\langle \log\left(p\left(y|w\right)p\left(w|u\right)\right)-\sum_{c}\gamma_{c}u_{c}^{2}-\sum_{c}\log\frac{1}{\gamma_{c}}\rangle }_{N\left(\theta |\mu_{\theta },\Sigma_{\theta }\right),G\left(\gamma_{i}|a_{i},b_{i}\right)|i=1\ldots C,i\neq j}\right),$$

with

$$\begin{array}{cc} {\langle \log(p(y|w)p(w|u))\rangle }_{N\left(\theta |\mu_{\theta },\Sigma_{\theta }\right),G\left(\gamma_{i}|a_{i},b_{i}\right)|i=1\ldots C,i\neq j} & =const.\;\mathrm{with}\;\mathrm{respect}\;\mathrm{to}\;\gamma_{j}\\ {\langle \sum_{c}\gamma_{c}u_{c}^{2}\rangle }_{N\left(\theta |\mu_{\theta },\Sigma_{\theta }\right),G\left(\gamma_{i}|a_{i},b_{i}\right)|i=1\ldots C,i\neq j} & =\left(\Sigma_{u_{j},u_{j}}+\mu_{u_{j}}^{2}\right)\gamma_{j}+const.\\ {\langle \sum_{c}\log\frac{1}{\gamma_{c}}\rangle }_{N\left(\theta |\mu_{\theta },\Sigma_{\theta }\right),G\left(\gamma_{i}|a_{i},b_{i}\right)|i=1\ldots C,i\neq j} & =const.\;\mathrm{with}\;\mathrm{respect}\;\mathrm{to}\;\gamma_{j}. \end{array}$$

This results in:

(27) $$\begin{array}{c} q_{\gamma_{j}}\left(\gamma_{j}\right)\leftarrow \frac{1}{\beta }exp\left(-\left(\Sigma_{u_{j},u_{j}}+\mu_{u_{j}}^{2}\right)\gamma_{j}\right)\propto G\left(\gamma |1,\left(\Sigma_{u_{j},u_{j}}+\mu_{u_{j}}^{2}\right)\right)\\ i.e.,\;a_{j}=1\;\mathrm{and}\;b_{j}\leftarrow \Sigma_{u_{j},u_{j}}+\mu_{u_{j}}^{2}. \end{array}$$

Ultimately, we can combine the two update rules in the following algorithm:

1. initialize ${\hat{\gamma }}_{i};\;i=1\ldots C$ with a low value
2. compute the mean and variance of $q_{\theta }\left(w,u\right)$ using Equations (24) and (25).
3. update $q(\gamma_{i})$, j=1,…,C from Equation (27).

By repeating steps 2 and 3 iteratively, we get our approximated solution of the posterior $q_{\theta }\left(w,u\right)$ and $q_{\gamma_{j}}\left(\gamma_{j}\right)$, j=1,…,C. At this point we would like to remark that one would get the same update rule following the Expectation Maximization (EM) algorithm. For more details we refer to the appendix in [30].

### 2.4. Uncertainty Map

For a autonomous exploration, we need a strategy that automatically determines sampling points that can be provided to a multi-robot system. To this end, we propose to use an uncertainty driven exploration strategy. We want to sample at locations where currently our knowledge about the gas concentration is more uncertain than in other regions. Therefore, we need a gauge for the uncertainty or the information content at each location in the environment. In a probabilistic or stochastic framework, there are different ways to quantify information content. For instance optimal experimental design for linear least square problems looks at the fisher information matrix of the unknown parameters [20,27,38]. Another option is to examine the confidence ellipsoid of a probability distribution, and based on this make conclusions about the parameter uncertainty [39]. More generally, the (Shannon) entropy can be considered to quantify the amount of information in a PDF [40]. However, all those concepts are closely related or just different interpretations of the same mathematical property. All concepts make use of the variance respectively covariance in one way or another. In optimal experimental design multiple properties of the covariance are considered, e.g., trace for A-optimality or determinant for D-optimality, etc. Considering the volume of the confidence ellipsoid, results in a similar criterion, since it is also proportional to the determinant of the covariance matrix. In the same way the entropy of a multi-variant normal distribution is calculated based on the determinant in the log domain.

As a conclusion: the second order moment is a feasible and often used gauge for the information content of a PDF. Thus, it is also used in this work. However, we are not interested in the information content of our complete joint PDF in Equation (11). Instead, we only want to compare the individual variables corresponding to the concentration values, associated with different regions in our environment. In order to get an individual PDF for each element in f, we thus calculate the marginal distributions. More precisely we calculate the marginals of the variational approximation of the posterior. Since only $q_{\theta }\left(w,u\right)$ contains information about f (remember the f is in w), we can find the marginals by:

(28) $$\begin{array}{c} p\left(f_{i}\right)=\int \ldots \int q_{\theta }\left(w,u\right)dw_{0}\ldots dw_{j-1}dw_{j+1}\ldots dw_{A}, \end{array}$$

where $w_{j}$ is the element in w that corresponds to $f_{i}$, i=1…C. Since $q_{\theta }\left(w,u\right)$ is a Gaussian distribution, the integration reduces to:

(29) $$p\left(f_{i}\right)\propto N\left(f_{i}|{\left[\mu_{w}\right]}_{j},{\left[\Sigma_{w,w}\right]}_{j,j}\right),$$

where ${\left[\mu_{w}\right]}_{j}$ is the element in $\mu_{w}$ corresponding to $f_{i}$ and ${\left[\Sigma_{w,w}\right]}_{j,j}$ the corresponding diagonal element in $\Sigma_{w,w}$. This variance is calculated for each element *i* in our state vector f. Furthermore, because of the discretization induced by the numerical approximation, each element in f can be associated with a discrete grid cell in a FVM approach or respectively mesh node in a FEM approach. In other words, each element has a spatial scope. Therefore also the elements of the covariance matrix that represent the uncertainty correlate to a spatial region and we can create the uncertainty map from them. Of course, this map is discretized according to the chosen numerical approximation.

Based on the uncertainty map, the design of our exploration strategy is straightforward. We send the agent of the multi-agent system for taking the next gas concentration measurements to locations with the highest uncertainty. More precisely, we select the *L* highest elements of ${\left[\Sigma_{w,w}\right]}_{j,j}$, i.e., highest uncertainty, and define the corresponding locations as POIs for the multi-agent system. Each agent moves to its assigned POI and takes a measurement there. After the next round of measurements has been collected, the uncertainty map is updated and the procedure starts again. At this point we want to note that while this procedure is quite simple from the conceptual perspective, a number of additional questions have to be considered for implementation purposes. This concerns for example, how to assign the POI or how to ensure that the agents do not collide. Those issues are beyond the scope of this paper and we would like to refer to our previous work [32] for further details.

## 3. Simulation and Results

A key contribution of this article is the evaluation of the influence of the proposed exploration algorithm in simulations. Our goal is to get an empirical understanding of the different parameters and to make sure that the proposed exploration strategy is able to find the gas sources. The finding in these simulations will put us in a position to parametrize our algorithm in real world scenarios in the future. In the next section, we first present the simulation setup used in more detail. After that we will present and discuss the results of the parameter evaluation.

### 3.1. Simulation Setup

Evaluating gas exploration strategies in real world experiments is known to be very challenging. Ground truth concentration values usually do not exist. It is also virtually impossible to design reproducible experiments with the exact same environment parameters, such as wind or source strength (i.e., gas release). Further, it is desirable to use realistic toy gases that are neither harmful to humans nor to the environment. With this in mind we decided to evaluate our exploration strategy using simulation. We use Equation (1) to forward simulate the gas dispersion. In this way we have full control over the wind condition and the number and location of sources. The simulated values at the sampling locations are used as point-wise synthetic measurements. We consider L=5 sampling locations (POI) in each iteration. The approach could thus be used to guide a multi-robot system consisting of five robots. Considerations regarding the robot dynamics and other constraints are out of the scope of this article. Here, we assume that the robots can reach every desired point in their environment within one iteration of our exploration algorithm (see Section 2.4). Further, the simulated gas concentration provides us with a ground truth value for calculating the error of our estimated gas distribution.

For the numerical calculation of the concentration field we use the same FEM approach as we used to generate Equation (4). Based on Equation (4) we can forward simulate the gas concentration f[n] with predefined $v_{1}\left[n\right]$, $v_{2}\left[n\right]$ and u[n]. To have a well posed problem additional boundary conditions are needed. In our case we decided for a Dirichlet boundary condition which corresponds to an open field scenario. As an initial condition we set f[0]=0 (all source start at time n=0). The matrices of Equation (4) are assembled based on the FEM software FENICS [33]. For our parameter evaluation, we assume a rectangular environment and have chosen to use a discretization mesh as a grid with 26x26 nodes. This results in 676 nodes, or 2304 unknowns in total for the numerical optimization at each time iteration of the exploration strategy (see Section 2.3). We estimate the concentration, source strength and two wind components at each node. We would like to remark that we treat the boundary condition as known in the model for the exploration. So the nodes at the border are not accounted as unknowns.

For gas simulation we consider a unit-less concentration and source strength. The diffusion coefficient κ in Equation (1) is set to 1 and the distance between two nodes in the mesh is also unit-less set to 1. By using these normalized values we reduce possible numerical issues. The time discretization Δt is part of the simulation studies. For some simulations we consider only the steady state of the PDE Equation (1). This means we set the time derivative to zero. From the implementation perspective this is achieved by setting $\frac{f[n]-f[n-1]}{\Delta t}=0$ in Equation (4).

**Figure 1:**
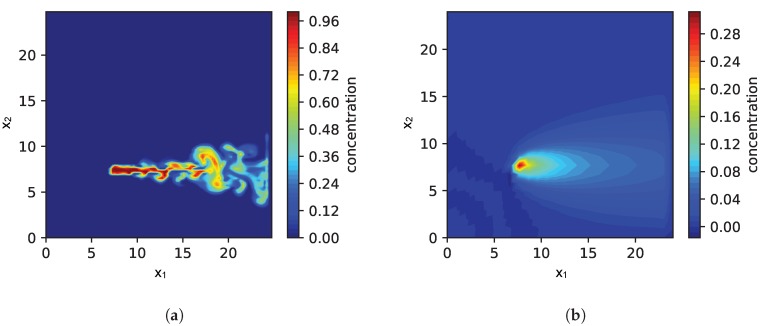
Comparison of the simulated gas concentration based on mantaflow in (**a**) and the PDE (1) in (**b**); While in (**b**) the space discretization is 26 × 26, in (**b**) the resolution of the simulation is 96 × 96 for a more accurate visualization.

With the simulation setup described so far, we are able to analyze the effect of model parameters such as the relaxation $\tau_{s}$, the influence of our sparse prior or wrong wind measurements. However, for simulating the environment we use exactly the same model as for the model-based exploration strategy. For evaluating the robustness of our approach regarding model mismatch, we need a different simulation that is not based on the PDE in Equation (1). We therefore used the fluid/smoke simulator mantaflow [41], which accounts for turbulence effects. The software is based on the Navier-Stokes equations and is able to simulate small scale turbulence and vortexes. However, the software tool primarily targets realistic smoke dispersion in the context of computer animations rather than gas dispersion. This does not matter so much for our purposes since we are interested in the effect of a model mismatch and therefore our intention is to use a simulation that differs from our model assumptions. As an example Figure 1 shows the instantaneous simulated gas distribution for both cases: (a) smoke simulation with mantaflow and (b) simulation based on PDE of Equation (1).

In this chapter we are going to evaluate the performance of the exploration strategy. To this end, we make use of two different error metrics which compare the estimates with the ground truth values. For the estimated gas concentration we use the Normalized Mean Square Error (NMSE), defined as: $\left|\right|\tilde{f[n]}-\hat{f}{\left[n\right]||}^{2}/\left|\right|\tilde{f}\left[n\right]{\left|\right|}^{2}$, where $\tilde{f}$ is the ground truth value and $\hat{f}$ the estimated concentration. Since we expect the estimated vector $\hat{u}$ to be sparse, we measure its difference to the ground truth $\tilde{u}$ by the Earth Mover’s Distance (EMD) [42]. EMD measures the effort needed to “displace” one distribution onto another one; it is thus particularly useful for comparing sparse functions. Essentially, it is the discrete equivalent to a Wasserstein metric.

### 3.2. Parameter Evaluation

#### 3.2.1. PDE Relaxation, Sensor Noise and Source Prior

The most important component studied in this article is the sparsity inducing prior for the source signals. A second important parameter of our approach is the relaxation of the model residuals parametrized by $\tau_{s}$. Actually, it is the ratio of this parameter to the measurement noise $\tau_{m}$ that plays an important role. From the results of the simulations we will see how the ratio influences the performance of the source localization. In order to gain insight into the importance of the sparsity assumption for source localization we carried out a set of simulations, where we used the PDE in Equation (1) to simulate the environment. We used a constant and uniform wind distribution with $v_{1}=1$ and $v_{2}=0$. Further, in this section we consider the steady state. For analyzing the influence of the prior, we averaged the performance of several simulation runs. In the individual runs we varied the number and the position of the sources, the relaxation parameter $\tau_{s}$ and the measurement noise $\tau_{m}$. More precisely, we carried out 45 simulations covering all possible combinations summarized in Table 1.

**Figure 2:**
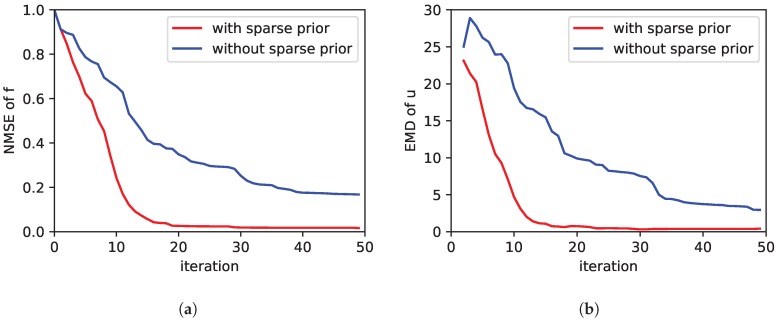
Comparison of the exploration performance: The two plots compare performance measures with and without the use of the sparsity assumption of the source distribution. In (**a**) the error of the estimated concentration field is shown as NMSE. In (**b**) the error of the estimated source distribution is plotted by means of the EMD. The curves are averaged over several simulation runs.

The results are shown in Figure 2. There we show the NMSE of the estimated concentration in Figure 2a and the EMD of the estimated source distribution in Figure 2b. The 45 simulation runs of the exploration were performed two times: once with sparsity inducing prior and once without the prior assumption. From the plot we can clearly see, that the performance of the exploration is better in case the sparse prior assumption is used. By “better” we mean the error is reduced faster, i.e., lower error values are achieved with less iterations. We consider 5 sampling points per iteration. Thus after each iteration five new measurements were added to the estimation problem. From the EMD plot in Figure 2b we can conclude that on average after 20 iterations the sources were correctly identified. From a numerical point of view, this means we were able to estimate the 576 elements of u with only 100 measurements.

Next we turn to the question how to choose the relaxation parameter $\tau_{s}$ and the measurement noise $\tau_{m}$. This time in the simulations we have chosen the parameters according to Table 2. The simulations cover all 125 possible combinations of parameters from this table.

**Figure 3:**
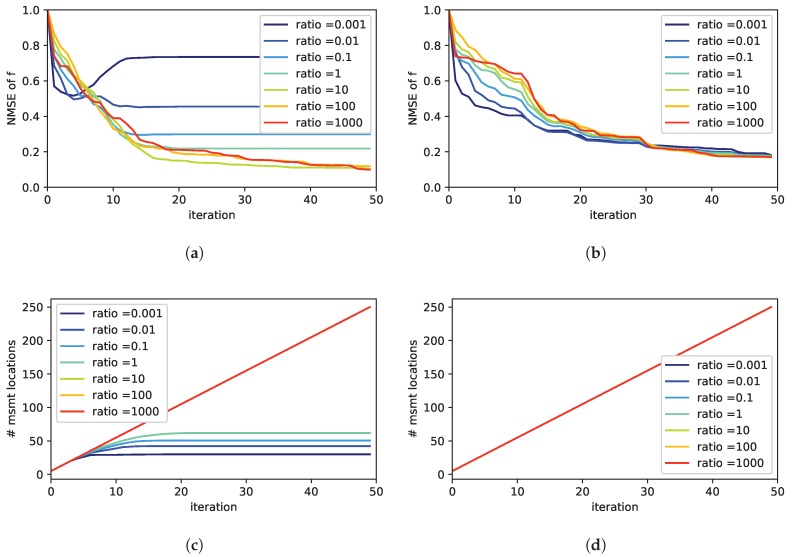
Effect of different ratios of $\tau_{m}/\tau_{s}$: On the left in (**a**,**c**) the case with a sparsity inducing prior is shown, on the right in (**b**,**d**) the case without. The first row (**a**,**b**) depicts the exploration performance by means of the NMSE of the estimated concentration field. The second row (**c**,**d**) plots the number of measurement locations. Note that in each iteration 5 measurements are carried out, however eventually at the same locations as before.

In Figure 3 we compare different ratios of $\tau_{m}/\tau_{s}$. We averaged over all simulation runs corresponding to a particular value of the ratio $\tau_{m}/\tau_{s}$. The plots in Figure 3a,b show the performance with respect to the NMSE. Once again, we compare the performance with (in Figure 3a) and without (in Figure 3b) the sparsity inducing prior. As can be seen, in the case no sparsity assumption is used, the performance is not as sensitive to the choice of $\tau_{m}$ and $\tau_{s}$ compared to the case with a sparse prior. Moreover, from Figure 3a it gets clear that a wrong choice may have a crucial impact on the exploration performance. In order to find out the reason for this, we looked more closely at the number of sampling locations. The number of measurement locations is shown in Figure 3c,d. Please note this is not the number of measurements. The number of measurements is increasing in every iteration by 5, i.e., the number of sampling points per iteration. However, some sampling points may be visited more than once. Therefore the number of measurement location not necessarily increases by 5 per iteration. As can be seen from Figure 3d, without a sparse prior the number of measurement location increases linearly by five for each iteration. This means that each new measurement is done at a new location that has not been visited before. If the sparse prior is used this is not always the case for all ratios of $\tau_{m}/\tau_{s}$. In Figure 3c the number of measurement locations converges for some ratios.

**Figure 4:**
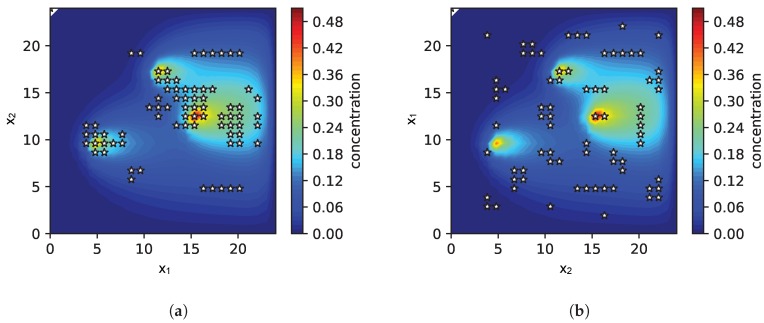
The two plots show as an example the sampling pattern for the strategy with a sparsity prior in (**a**) and without the use of a sparsity prior in (**b**). The parameters were chosen as $\tau_{m}$ = $10^{5}$, $\tau_{s}$ = $10^{4}$. The white stars indicate the measurement locations. The color map represents the ground truth gas concentration field. The sources are obviously located at the locations with the highest concentration. In both cases the scene is a snapshot after 15 iterations.

When looking into the sampling pattern of the exploration strategy more closely in Figure 4, we found out that the exploration strategy prefers regions close to potential sources in case when the sparsity prior is used.

This pattern is the reason for the successful and faster identification of the exact source distribution. In contrast, without a sparse prior, the samples are more or less equally distributed over the environment as can be seen in Figure 4b.

A possible intuitive explanation of this behavior is as follows. The presence of an estimated source implies a violation of the prior assumption, which assumes that only few sources are distributed in the environment. Every new source slightly contradicts the sparsity assumption. As a consequence, the uncertainty in the respective region grows and it gets more favorable for further measurements. In general, this is not a problem since the new measurements in the region automatically reduce the uncertainty and compensate this effect. However, in case of low ratios of $\tau_{m}/\tau_{s}$, the trust in the measurements is comparably low. In other words, sensor noise is high and measurements are not considered informative. Therefore, the measurements are not able to compensate for the effect of the sparsity inducing prior. As a consequence, the exploration strategy will tend to demand measurements always at the same locations. Thus, the number of measurement locations does not increase after some time; Instead, measurements are repeated in the same regions and it is difficult to find the true source distribution. From the different simulations we found that a ratio of $\tau_{m}/\tau_{s}=10$ seems to be a good choice. Further, we found the best exploration performance for the combination $\tau_{m}$ = $10^{5}$ and $\tau_{s}$ = $10^{4}$.

#### 3.2.2. Wind Prior

In this article, we consider the wind field as a priori known. It is conveniently plugged into the probabilistic formulation by a prior for the wind field components of Equation (9). This prior could be given by some forecast or recent measurements in the environment. However, what if the prior is wrong? To analyze this question we carried out 181 simulations with the parameter combinations summarized in Table 3. Once again we stick to the static steady state for an easier evaluation. Further, we consider only a single source at point (10,10). The precision for $\tau_{m}$ and $\tau_{s}$ was chosen according to the findings of the previous section.

**Figure 5:**
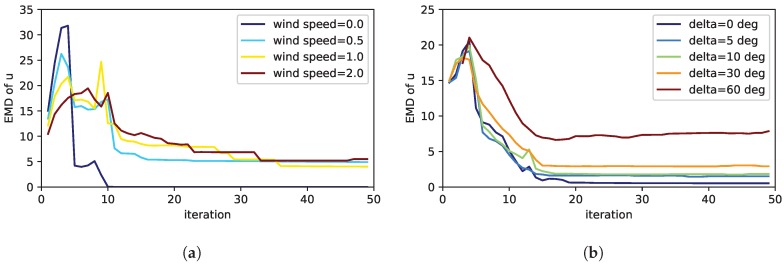
Analysis of a wrong wind prior on the performance of the exploration strategy measured by the EMD of the estimated source distribution: In (**a**) the exploration strategy was informed by the wind prior that there is no wind. The different curves show the performance for different simulated wind speeds contradicting the wind prior. Similarly (**b**) depicts the case, when the assumption about the wind speed encoded in the wind prior of the exploration strategy coincides with the simulation, but the wind direction differs between the actual simulation and the prior assumption. For the sparsity prior, relaxation parameter and measurement noise we have chosen the best values found in the previous section.

**Figure 6:**
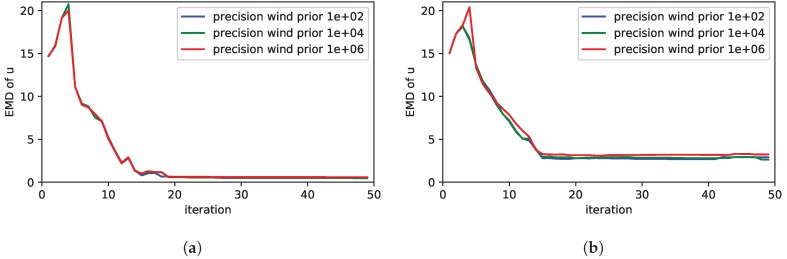
Studies of the precision of the wind prior $\tau_{v}$ on the performance of the exploration strategy measured by the EMD of the estimated source distribution: In (**a**) the prior of the wind matches with the simulated wind, in (**b**) the wind direction differs by 30 deg.

Figure 5a shows the results for the case the prior wind field claims that there is no wind. As long as the true simulated wind speed is zero, the performance of the exploration is good. However, as soon as the simulated true wind speed is higher than 0, the performance decreases due to the wrong prior assumptions. This shows the importance to take wind into account in the model for the exploration. Thus a advection-diffusion model is of advantage compared to a pure diffusion PDE. In Figure 5b we studied the case where the wind direction of the prior differs from the simulated wind. As can be seen, this also affects the exploration performance. However, the performance is still acceptable up to an error of 30 deg in the wind direction. Moreover, the question arises how to set the precision $\tau_{v}$ for the wind prior. To answer this question, we first varied the precision for the prior in a case where the prior wind speed and direction match with the simulation. In this case, as Figure 6a shows, selecting different values of the wind precision does not have a noticeable effect on the exploration performance. In a second step we analyzed the impact of the precision in case the wind prior does not match with the wind in simulation. In particular Figure 6b shows the situation where the wind direction of the prior and the ground truth differs by 30 deg. Even in this case, the effect of setting different values of the wind precision is largely negligible. We only noticed a very small advantage, if a low precision was chosen in case of a wrong wind prior.

#### 3.2.3. Time Discretization

**Figure 7:**
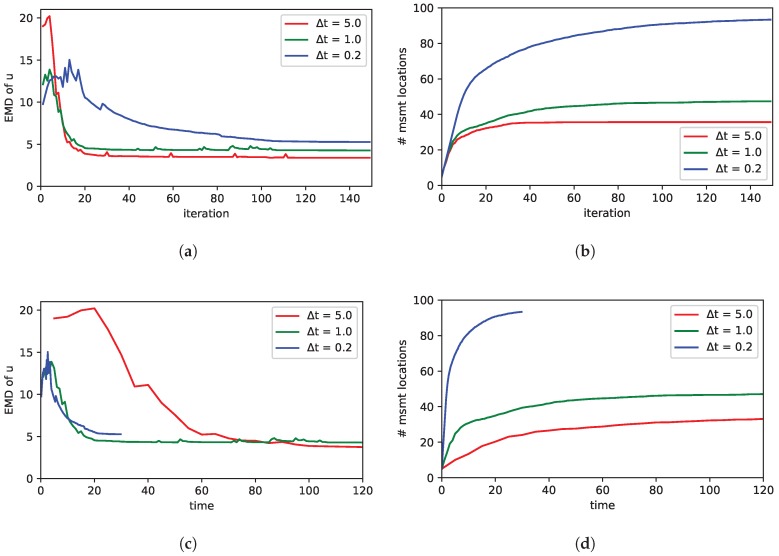
Comparison of different values of Δt, i.e., the time discretization of the PDE (note that Δt can be interpreted as the inverse sampling rate): The plots in (**a**) show the performance of the exploration measured by the error in the estimated source distribution by the EMD. In (**b**) the number of measurement locations is shown. Note that in each iteration 5 measurements are carried out, however measurements may be carried out at the same locations. The plots in (**c**,**d**) show the same data, but the x-axis is scaled according to time, not iterations.

So far we have considered only the steady state of our dynamic process. In this section we analyze the effect of the chosen time discretization in the numerical approximation of the PDE. Essentially, we examine the performance of the exploration for different values of Δt in Equation (4), still performing 5 measurements in each iteration. So actually, Δt describes the inverse sampling frequency of our measurements. It is the effect of this sampling frequency that we would like to analyze here. For this purpose we ran multiple simulations with all 90 combinations of parameters shown in Table 4. Apart from that we have chosen a constant wind field, which is perfectly known by the prior with $\tau_{v}$ = $10^{5}$. In the dynamical case, additionally, an initial condition for the PDE is required. We have chosen the concentration field to be zero (f[0]=0) at the start of a simulation run. The results are averaged over the different combinations of parameters for each fixed value of Δt. Figure 7 shows the results. Analyzing and interpreting these results is quite difficult because different effects play a role and they cannot be studied separately.

First, let us consider what it means to increase Δt. Higher values correspond to more time in between measurements. So the dynamic process is faster or more dynamic compared to the sampling frequency. In other words, the gas spreads out faster with respect to the number of measurement iterations as in case of Figure 7. In contrast, a low Δt means that a lot of measurement iterations pass until the gas is spread out. Thus in case of a low Δt, gas is mostly concentrated around the source and not widely spread during the first iterations. This makes it more difficult to find the sources. This effect may explain the fact that the performance of the exploration with a higher Δt is better considering the number of iterations; as can be seen in Figure 7a. Here better means: fewer measurements (i.e., iterations) are needed. However, if absolute time in Figure 7c is considered, with a lower Δt (i.e., higher sampling rate) the error converges faster. Of course in case of a lower Δt, also more measurements are collected with respect to time (see Figure 7d.)

Another effect that has to be considered in the dynamic case is aging of measurements. Because of the iterative update of the model in each time stamp *n*, the influence of a preceding measurement decreases over time. Note that previous measurements are indirectly encoded through the term f[n−1] in (4). If the time between two update iterations is higher (i.e., higher Δt), this aging or forgetting of measurements has a stronger impact. As a consequence, the exploration strategy tends to repeat measurements at locations, which have been measured already in the past for higher values of Δt. This effect can be seen in Figure 7b. Moreover, we would like to remark that also the identification of sources in combination with the sparsity inducing prior may influence the saturation of the number of measurement locations as discussed in Section 3.2.1. However, it is hard to separate those two effects.

#### 3.2.4. Dispersion Model Mismatch

Finally, we are interested in the effect of a model mismatch on the performance of the source localization in case the gas dispersion is simulated differently. Instead of using the same PDE for forward-simulation and the exploration strategy, we simulate the gas dispersion using the mantaflow smoke simulator. The mantaflow smoke simulator models more realistic flow dynamics and allows us thus to observe a situation that we expect also in real-world applications. Our approach is based on a simplified model of fluid flow (the PDE in Equation (2)). It models important components of fluid flow, but not all its complexity. Using a simplified model allows us to exploit domain knowledge and to constrain the computational complexity but, of course, it is important to know what are the consequences of this model mismatch. In order to address this question, we simulated a single source. An exemplary snapshot of the instantaneous concentration field is shown in Figure 1a. Measurements taken during exploration are averaged over 20 s. In this way we take the typically slow sensor response of gas sensors into account. Please note that, as a side effect, this smooths out rapid fluctuations caused by turbulence in the gas dispersion. The PDE process model used in the exploration is parametrized as follows: The wind prior was set to 2 for the component in $x_{1}$-direction. The precision $\tau_{s}$ was selected from $10^{3}$, $10^{4}$, $10^{5}$ while the ratio $\tau_{m}/\tau_{s}=10$ was fixed. Moreover, we approximate the gas dispersion just with the steady state of the PDE.

**Figure 8:**
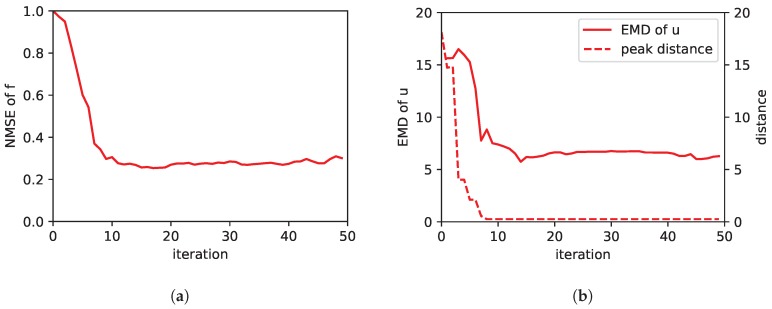
Performance of our exploration strategy in case of the mantaflow simulation: the plot in (**a**) shows the NMSE of the estimated concentration field. In (**b**) the EMD of the estimated source distribution is plotted. Besides, the Euclidean distance of the location of the simulated source and the peak in the estimated source distribution is shown.

**Figure 9:**
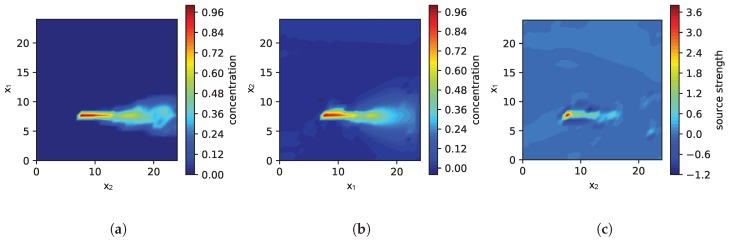
Exploration of a gas distribution simulated with mantaflow after 10 iterations (50 measurements): Plot (**a**) shows a snapshot of the instantaneous gas concentration field simulated by mantaflow. In (**b**) the estimated concentration field is shown and (**c**) depicts the estimated source distribution.

The results are shown in Figure 8. In general, the performance of the estimate is worse compared to the results without model mismatch, as to be expected. Both, the NMSE of the estimated concentration and the EMD of the estimated source distribution is high. To illustrate how good or bad these values are, we plotted a snapshot of the estimation during the exploration in Figure 9. In this case we have chosen $\tau_{s}$ = $10^{4}$ and $\tau_{m}$ = $10^{5}$ and the snapshot was taken after 10 iterations (i.e., 50 measurements). Figure 9a shows the concentration field averaged over 20 s. Values from this field are used as measurements in the simulation. Figure 9b depicts the estimated concentration field and Figure 9c the estimated source distribution, respectively. While we would expect the source distribution to be a single peak, it can be seen that the actual estimated source distribution is a wider shaped distribution. This is the reason for the bad EMD value. However, in this simple case with a single source we can also calculate the distance of the maximum of the source distribution (peak) as the most likely location of the source and the ground truth location of the source. This is plotted additionally in Figure 8b. There we see that despite the shape of the source distribution is wrong, the location of the source is correct.

To sum up, we observed a certain drop in the performance of the estimate of the source distribution based on the exploration strategy when we change to the mantaflow simulator. In general we would expect a similar behavior in a real-world application. Whether the estimated location of the source is good enough to claim that the sources were successfully found, depends on a particular application.

## 4. Conclusions

In this paper, we propose a model-based exploration strategy for a multi-robot system. The purpose of the exploration strategy is to guide robots equipped with gas sensors to informative measurements locations. These concentration measurements help to infer and localize unknown gas sources in the environment of the robots. We have shown that it is possible to incorporate physical knowledge about the gas dispersion process. In this article, we introduce as a mathematical model an advection-diffusion PDE to describe the gas dispersion. Our approach facilitates the integration of additional prior information. For example, we consider the sources to be sparsely distributed in the environment. In other words, we treat the number of sources as unknown. This is a weaker assumption, compared to previous approaches which require knowledge about the exact number of sources. We would like to remark that even though the objective of our approach is gas source localization, the approach provides also a map of the gas concentration distribution as a side product. This is maybe of advantage in disaster scenarios, where the task is also to eliminate the gas sources. There the map can be used to plan a path towards the source that avoids to expose rescue staff or material to high contamination.

In this paper the performance of the proposed exploration strategy was evaluated in different simulations. The simulations allowed us to extensively study the different parameters of the presented algorithm as well as effects such as errors in the prior assumptions, e.g., concerning the wind. The key findings of the simulations should be considered in the future, when applying the approach to real world scenarios. We have found that the sparsity assumption about the source distribution has a significant impact on the performance of the exploration strategy. Compared to the case where the sparsity assumption is not used, a better estimate of the source distribution is achieved faster by incorporating the sparsity assumption into the exploration strategy. Moreover, we investigated the effect of the integrated advection term. We studied whether there could be, for example, even negative effects if wind information is “too” wrong—and what “too wrong” means. We found that it is possible to find the sources even if airflow is (falsely) ignored. However, the estimation is worse and the exploration takes longer compared to when sufficiently correct the wind information is incorporated. Whether the performance is sufficient has to be answered with respect to a certain application and depending on the quality of wind information. Wrong a priori wind information is tolerable to a certain extent. Again, making a categorical statement is difficult without a particular application in mind. Our results suggest that the wind estimate should be accurate to at least 30 deg. In addition, we evaluated the exploration in a simulation with data generated using a model that differs significantly from the dispersion model used in the exploration strategy. The performance achieved in those simulations is closer to what can be expected in real world scenarios. Due to the model mismatch, the quality of the estimated source distribution decreased. The source was no longer clearly estimated as a single narrow peak, but as a broader shaped distribution. Nevertheless, the location of the peak of the estimated distribution was found to coincide with the actual source location.

Even though we analyzed a number of different effects on the exploration performance, some question remain for future work. This concerns first the impact of numerical issues on the presented approach remains. For example the impact of different space discretizations of the FEM needs some further investigation. Here an interesting question is: how does a higher resolution (i.e., finer discretization) of the source distribution influence the required exploration time and performance. Another open challenge is to compare the proposed exploration strategy to a meaningful benchmark algorithm. As mentioned in the introduction, the authors are not aware of another, adaptive gas source localization approach that can cope with an unknown number of sources. A comparison with strategies that use predefined trajectories (e.g., a sweeping trajectory, random sampling etc.) is also difficult and often unfair. The performance of those strategies highly depends on the particular situations considered and the location of gas sources. For example with a random sampling strategy a lucky run may find the sources comparably fast in some runs while other runs may perform extremely badly. Therefore, we think the most reasonable and also challenging benchmark performance in the future would be to compare to a trained human operator. Last but not least, in the future, we will apply the proposed algorithm to real world experiments. For this purpose we already started to develop a suitable robotic platform and tested a similar algorithm in hardware-in-the-loop experiments [43]. In the presented article, we incorporated wind information as a given a-priori known wind field map. In practice, modeling airflow and creating such a wind field map is not fully solved yet, but an active research field. A possible solution would be to fuse the presented approach with probabilistic wind field maps as in [34].

## Figures and Tables

**Table 1:** Parameters used for simulation runs shown in Figure 2.

Source(s) at	(10,10); (15,13); (10,17), (17,7); (8,12), (15,12); (12,17), (5,10), (15,12)
$\tau_{s}$	$10^{4}$ ; $10^{5}$ ; $10^{6}$
$\tau_{m}$	$10^{4}$ ; $10^{5}$ ; $10^{6}$

**Table 2:** Parameters used for simulation runs shown in Figure 3.

Source(s) At	(10,10); (15,13); (10,17), (17,7); (8,12), (15,12); (12,17), (5,10), (15,12)
$\tau_{s}$	$10^{2}$ ; $10^{3}$ ; $10^{4}$ ; $10^{5}$ ; $10^{6}$
$\tau_{m}$	$10^{2}$ ; $10^{3}$ ; $10^{4}$ ; $10^{5}$ ; $10^{6}$

**Table 3:** Parameters used for analyzing the influence of the wind prior (Note: for the case prior wind speed 0 the wind direction was not varied).

prior wind speed	0 ; 0.5 ; 1 ; 2
prior wind direction	0 deg ; 5 deg ; 10 deg ; 30 deg ; 60 deg
$\tau_{v}$	$10^{2}$ ; $10^{4}$ ; $10^{6}$
ground truth wind speed (direction 0 deg)	0 ; 0.5 ; 1 ; 2

**Table 4:** Parameters used for simulation runs shown in Figure 7.

Source(s) At	(10,10); (15,13); (10,17), (17,7); (8,12), (15,12); (12,17), (5,10), (15,12)
$\tau_{s}$	$10^{3}$ ; $10^{4}$ ; $10^{5}$
$\tau_{m}$	$10^{5}$ ; $10^{6}$
delta *t*	0.2 ; 1.0 ; 5.0

## Abbreviations

The following abbreviations are used in this manuscript:

PDE	Partial Differential Equation
POI	Point of Interest
PDF	Probability Density Function
FEM	Finite Element Method
FVM	Finite Volume Method
SBL	Sparse Bayesian Learning
UAV	Unmanned Aerial Vehicle
EMD	Earth Mover’s Distance
EM	Expectation Maximization
CBRN	chemical, biological, radiological and nuclear
NMSE	Normalized Mean Square Error
